# Supplementary material for: Macroscale intrinsic dynamics are associated with microcircuit function in focal and generalized epilepsies
Source: Commun Biol. 2024 Feb 1;7:145. doi: 10.1038/s42003-024-05819-0 (PMC10834476; doi:10.1038/s42003-024-05819-0)
Supplement: Supplementary file 2 — Supplementary Information [file 42003_2024_5819_MOESM2_ESM.pdf]

Supplementary Information for:

Macroscale Intrinsic Dynamics are associated with Microcircuit Function in Focal and Generalized Epilepsies

Siqi Yang[1,2,3], Yimin Zhou[1], Yao Meng[2], Huafu Chen[2], Shaoshi Zhang[3], Xiaolu Kong[3], Ru Kong[3], B.T Thomas Yeo[3], Wei Liao[2]✉, Zhiqiang Zhang[4]✉

[1] School of Cybersecurity (Xin Gu Industrial College), Chengdu University of Information Technology, Chengdu, 610225, P.R. China.
[2] The Clinical Hospital of Chengdu Brain Science Institute, School of Life Science and Technology, University of Electronic Science and Technology of China, Chengdu, 610054, P.R. China.
[3] Centre for Sleep and Cognition (CSC) & Centre for Translational Magnetic Resonance Research (TMR), Yong Loo Lin School of Medicine, National University of Singapore, Singapore, Singapore.
[4] Lab. of Neuroimaging, Dept. of Radiology, Jinling Hospital, Nanjing University School of Medicine, Nanjing, 210002, P.R. China.

✉ Corresponding authors:
Zhiqiang Zhang (zhangzq2001@126.com)
Lab. of Neuroimaging, Dept. of Radiology, Nanjing Jinling Hospital, 305#, Eastern Zhongshan Rd., Nanjing 210002, P.R. China. Fax: +86-25-84804659. Tel: +86-25-84804659.

And Wei Liao (weiliao.wl@gmail.com)
The Clinical Hospital of Chengdu Brain Science Institute, MOE Key Laboratory for Neuroinformation, University of Electronic Science and Technology of China, Chengdu 611731, P.R. China. Fax: +86-28-61831273. Tel: +86-28-61831273.

CONTENTS

Supplemental Tables 3

Supplemental Figures 4

Supplemental Table

Supplementary Table 1 Demographic and clinical characteristics of participants in training, validation and test sets.

	Training set				Validation set				Test set			
	HC (n=41)	TLE (n=23)	GTCS (n=26)	*P*-value	HC（n=37）	TLE（n=28）	GTCS（n=27）	*P-value*	HC（n=30）	TLE（n=24）	GTCS（n=26）	*P-value*
Sex (males/females)	22/19	12/11	18/8	0.37[b]	18/19	15/13	18/9	0.35[b]	18/19	15/13	18/9	0.72[b]
handedness (left/right)	0/41	0/23	0/26	1	0/37	0/28	0/27	1	0/30	0/24	0/26	1
Age (years)	23.88 ± 5.27	25.26 ± 7.73	24.69 ± 7.65	0.80[a]	23.30 ±2.50	27.36 ±10.63	23.89 ±8.44	0.13[a]	24.03 ±4.14	24.25 ±6.69	25.81 ±7.56	0.89[a]
Duration of illness (months)	—	141.60± 90.63	76.81± 70.11		—	105.00 ± 89.83	90.30 ± 121.70		—	91.50 ± 83.21	94.58 ± 83.10	

Note： Values are mean ± standard deviation (SD).

Abbreviations: HC, healthy controls; TLE, temporal lobe epilepsy; GTCS, genetic generalized epilepsy with generalized tonic-clonic seizures.

[a] represented that P-value was obtained by Kruskal-Wallis test；

[b] represented that P-value was obtained by $\chi 2$ test.

Supplemental Figures

Supplementary Figure 1. The significant difference in recurrent connection (*RC)* between epilepsies and controls.

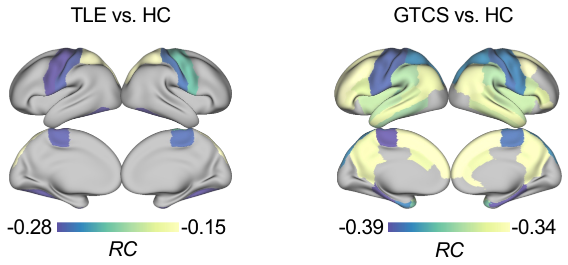

To determine the region with significantly statistical changes, a total of 1,000 permutations ($P_{FDR} < 0.05$) with randomly shuffling the epilepsies and controls within training, validation and test set, and then repeated the analysis in the pMFM procedure.

Supplementary Figure 2. The spatial pattern of the simulated PC1 and PC2 in each group.

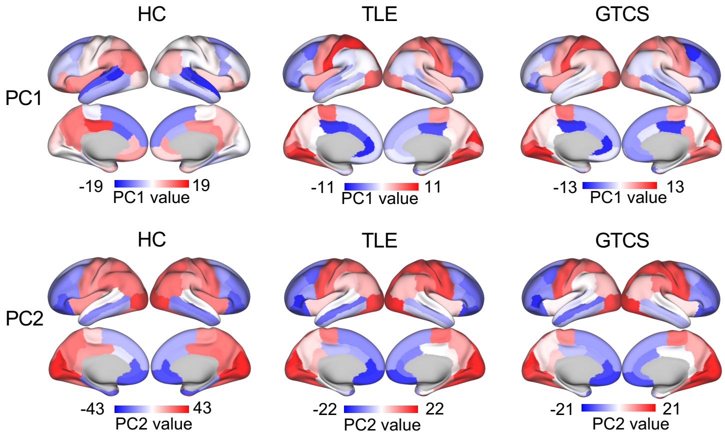

In each group, massive time-series features of BOLD fMRI simulated from the pMFM was extracted using *hctsa* toolbox, and the gradients (PC1 and PC2) was computed using PCA technique.

Supplementary Figure 3. Results of re-analysis using a higher resolution atlas with Schaefer 200 cortical regions.

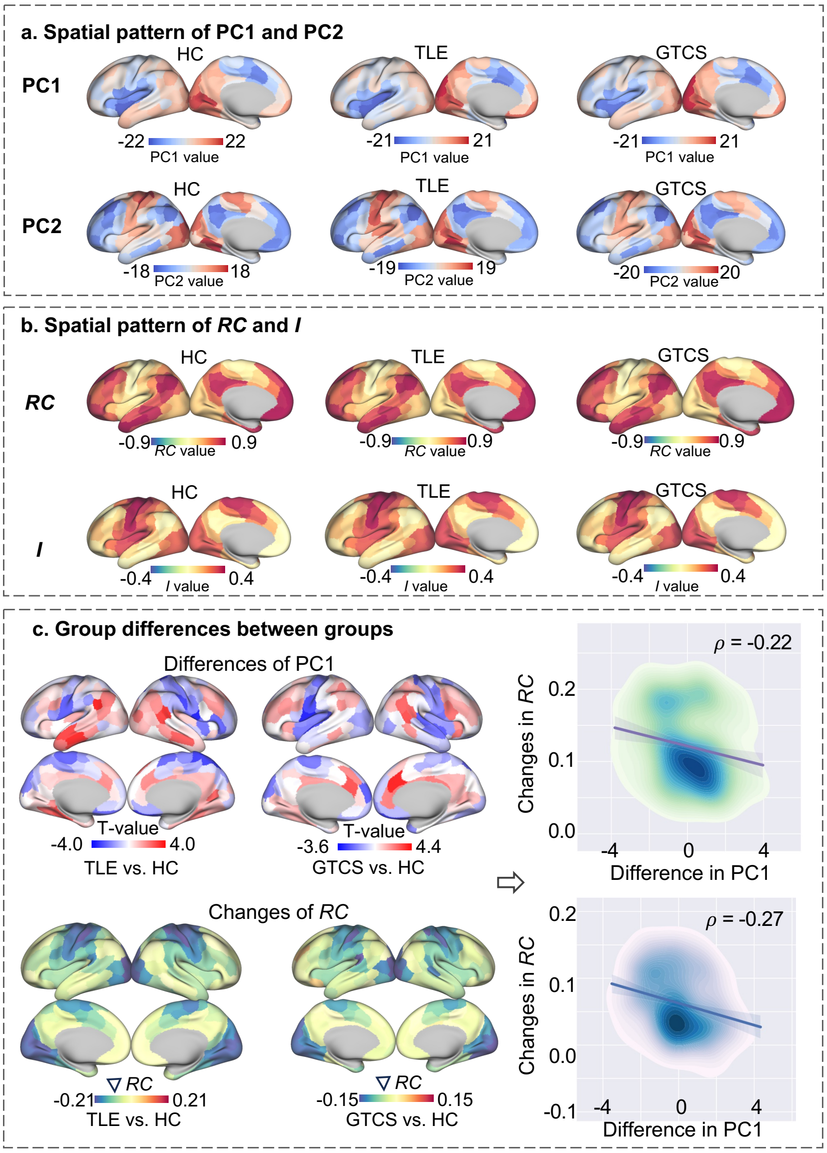

With a higher resolution parcellation with Schaefer 200 cortical regions, spatial pattern of the PC1 and PC2 (**a**), and the *RC* and *I* (**b**). Network-wise differences in PC1 and PC2 were compared (**b**). Associations between the PC1 and *RC* in two epileptic subtypes (**c**). All p < 0.05 with Spearman correlation, n = 200 represented number of brain parcels. The distribution of observations in each brain parcel used a kernel density estimate method.

Supplementary Figure 4. Macroscale intrinsic dynamics in subcortical regions.

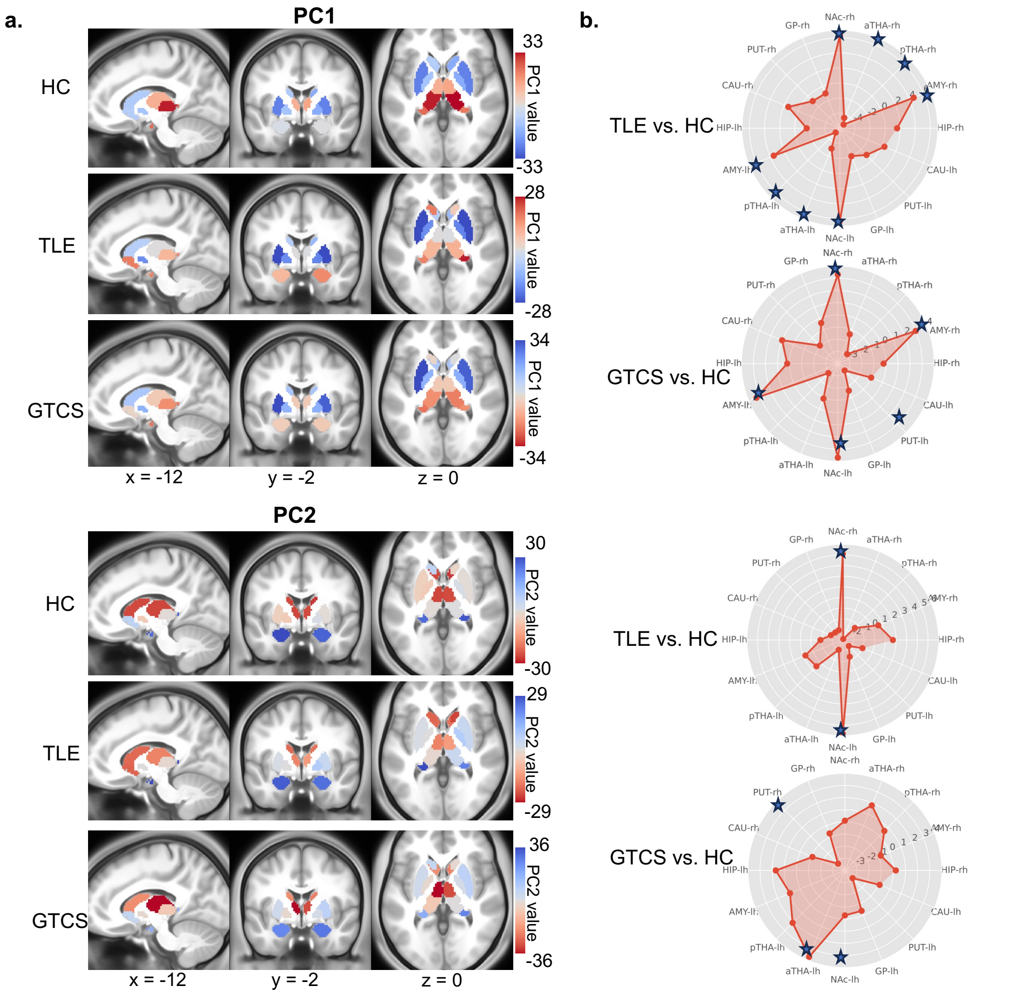

Spatial pattern of the PC1 and PC2 in each group after Procrustes alignment (**a**). Network-wise differences in PC1 and PC2 were compared (**b**). A radar plot shows the difference between the epilepsies (TLE and GTCS) and HC. The blue star represents significant difference between groups, $P < 0.05/16$ with Bonferroni correction. The red line represents the two-sample *t*-test statistical values between groups.

Supplementary Figure 5. Two simple time series properties and macroscale intrinsic dynamics.

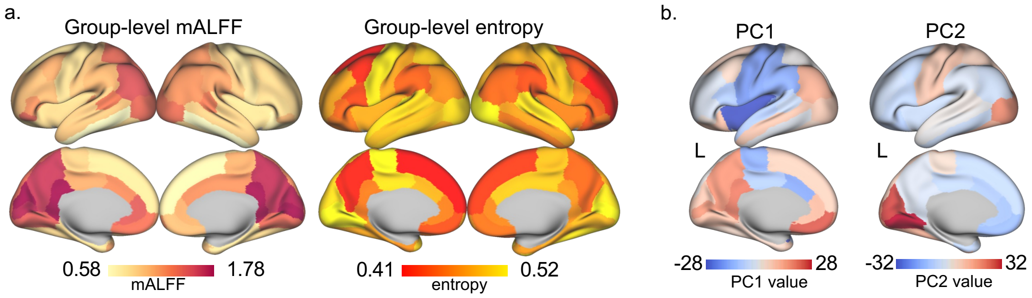

The spatial pattern of the group-level mALFF (amplitude of low-frequency fluctuation) and temporal entropy (**a**) and the intrinsic dynamics gradients (PC 1 and PC2) (**b**).
