# Supplementary material for: Macroscale intrinsic dynamics are associated with microcircuit function in focal and generalized epilepsies
Source: Commun Biol. 2024 Feb 1;7:145. doi: 10.1038/s42003-024-05819-0 (PMC10834476; doi:10.1038/s42003-024-05819-0)
Supplement: Supplementary file 3 — Description of Additional Supplementary Files [file 42003_2024_5819_MOESM3_ESM.pdf]

Description of Additional Supplementary Files

File name: Supplementary Data 1
Description: The source data used to plot Figure 2c.

File name: Supplementary Data 2
Description: The source data used to plot Figure 5.
