# Supplementary material for: Macroscale intrinsic dynamics are associated with microcircuit function in focal and generalized epilepsies
Source: Commun Biol. 2024 Feb 1;7:145. doi: 10.1038/s42003-024-05819-0 (PMC10834476; doi:10.1038/s42003-024-05819-0)
Supplement: Supplementary file 6 — Reporting Summary [file 42003_2024_5819_MOESM6_ESM.pdf]

nature portfolio

Corresponding author(s): Zhiqiang Zhang

Last updated by author(s): Jan 10, 2024

Reporting Summary

Nature Portfolio wishes to improve the reproducibility of the work that we publish. This form provides structure for consistency and transparency in reporting. For further information on Nature Portfolio policies, see our Editorial Policies and the Editorial Policy Checklist.

Statistics

For all statistical analyses, confirm that the following items are present in the figure legend, table legend, main text, or Methods section.

n/a	Confirmed	
☐	☒	The exact sample size (*n*) for each experimental group/condition, given as a discrete number and unit of measurement
☐	☒	A statement on whether measurements were taken from distinct samples or whether the same sample was measured repeatedly
☐	☒	The statistical test(s) used AND whether they are one- or two-sided<br>*Only common tests should be described solely by name; describe more complex techniques in the Methods section.*
☐	☒	A description of all covariates tested
☐	☒	A description of any assumptions or corrections, such as tests of normality and adjustment for multiple comparisons
☐	☒	A full description of the statistical parameters including central tendency (e.g. means) or other basic estimates (e.g. regression coefficient) AND variation (e.g. standard deviation) or associated estimates of uncertainty (e.g. confidence intervals)
☐	☒	For null hypothesis testing, the test statistic (e.g. *F*, *t*, *r*) with confidence intervals, effect sizes, degrees of freedom and *P* value noted<br>*Give P values as exact values whenever suitable.*
☒	☐	For Bayesian analysis, information on the choice of priors and Markov chain Monte Carlo settings
☒	☐	For hierarchical and complex designs, identification of the appropriate level for tests and full reporting of outcomes
☐	☒	Estimates of effect sizes (e.g. Cohen's *d*, Pearson's *r*), indicating how they were calculated

Our web collection on statistics for biologists contains articles on many of the points above.

Software and code

Policy information about availability of computer code

Data collection	No software was used for data collection.
Data analysis	Functional images were preprocessed using the DPABI toolbox (http://http://rfmri.org/dpabi). Then structural connectomes were generated from preprocessed DWI using MRtrix3 (https://github.com/MRtrix3/mrtrix3).<br>The code of pMFM was referred: https://github.com/ThomasYeoLab/CBIG/tree/master/stable_projects/fMRI_dynamics/Kong2021_pMFM;<br>The hctsa toolkit was downloaded from: https://github.com/benfulcher/hctsa.

For manuscripts utilizing custom algorithms or software that are central to the research but not yet described in published literature, software must be made available to editors and reviewers. We strongly encourage code deposition in a community repository (e.g. GitHub). See the Nature Portfolio guidelines for submitting code & software for further information.

Data

Policy information about availability of data

All manuscripts must include a data availability statement. This statement should provide the following information, where applicable:

- Accession codes, unique identifiers, or web links for publicly available datasets
- A description of any restrictions on data availability
- For clinical datasets or third party data, please ensure that the statement adheres to our policy

The data that support the findings of this study are available on request from the corresponding author.

Research involving human participants, their data, or biological material

Policy information about studies with human participants or human data. See also policy information about sex, gender (identity/presentation), and sexual orientation and race, ethnicity and racism.

Reporting on sex and gender	Sex effects was not considered in this study.
Reporting on race, ethnicity, or other socially relevant groupings	N/A.
Population characteristics	Age and sex were regressed out.
Recruitment	Two types of patients had negative presentation on diagnostic MRI (i.e., no lesion such as cortical dysplasia, benign brain tumor or hippocampal sclerosis was detected), and none had received surgical treatment up to time of data preparation for this work. According to International League against Epilepsy (ILAE) classifications46, epilepsies were diagnosed by two experienced neurologists. For patients with TLE, the patients present with (a) typical symptoms of TLE, such as automatism (hand, oral), autonomic symptoms, olfactory hallucination, and complex partial seizures with aura; and (b) specific patterns of electrophysiological activity recorded by scalp EEG, such as epileptic spikes in the bilateral frontotemporal or temporal lobes. For genetic generalized epilepsy with GTCS, the patients presented with a) typical seizure semiology of GTCS, including loss of consciousness during seizures without precursory symptoms of partial epilepsy and aura, and tonic extension of the limbs followed by a clonic phase of rhythmic jerking, b) generalized spike-and-wave discharges on EEG, and c) no other epilepsy associated etiology such as trauma, tumor, or intracranial infection.
Ethics oversight	All study protocols were performed according to the Helsinki Declaration of 1975 and approved by the medical ethics committee of Jinling Hospital, School of Medicine, Nanjing University.

Note that full information on the approval of the study protocol must also be provided in the manuscript.

Field-specific reporting

Please select the one below that is the best fit for your research. If you are not sure, read the appropriate sections before making your selection.

☒ Life sciences ☐ Behavioural & social sciences ☐ Ecological, evolutionary & environmental sciences

For a reference copy of the document with all sections, see nature.com/documents/nr-reporting-summary-flat.pdf

Life sciences study design

All studies must disclose on these points even when the disclosure is negative.

Sample size	We included two epileptic subtypes: patients with temporal lobe epilepsy (TLE, n = 75) and patients with genetic generalized epilepsy with tonic-clonic seizures (GTCS, n = 79), as well as sex-, age-matched healthy controls (HC, n = 108).
Data exclusions	Patients were excluded for i) progressive diseases, malformations of cortical development, tumors, or previous neurosurgery, iii) incomplete MR scanning, or iv) excessive head motion during scanning.
Replication	The HC, TLE and GTCS groups were divided into three subsets: training set, validation set and test set.
Randomization	No randomization is performed.
Blinding	No blinding is done.

Reporting for specific materials, systems and methods

We require information from authors about some types of materials, experimental systems and methods used in many studies. Here, indicate whether each material, system or method listed is relevant to your study. If you are not sure if a list item applies to your research, read the appropriate section before selecting a response.

Materials & experimental systems

n/a	Involved in the study
☒	☐ Antibodies
☒	☐ Eukaryotic cell lines
☒	☐ Palaeontology and archaeology
☒	☐ Animals and other organisms
☒	☐ Clinical data
☒	☐ Dual use research of concern
☒	☐ Plants

Methods

n/a	Involved in the study
☒	☐ ChIP-seq
☒	☐ Flow cytometry
☐	☒ MRI-based neuroimaging

Plants

Seed stocks: N/A

Novel plant genotypes: N/A

Authentication: N/A

Magnetic resonance imaging

Experimental design

Design type: Resting-state fMRI

Design specifications: not relevant

Behavioral performance measures: nor relevant

Acquisition

Imaging type(s): Functional, structural, and diffusion.

Field strength: 3 Tesla

Sequence & imaging parameters: Functional MR images (fMRI) were acquired using an echo-planar sequence (repetition time = 2000 ms, echo time = 30 ms, and flip angle = 90°). Thirty-three transverse slices (field of view = 240 × 240 mm2, matrix = 64 × 64, slice thickness = 4 mm, and interslice gap = 0.4 mm) aligned along the anterior commissure–posterior commissure line was acquired with a total of 250 volumes. Total scan time was 500 s. T1-weighted (T1w) MRI were acquired in a sagittal orientation using a magnetization-prepared rapid gradient-echo sequence (repetition time (TR)/echo time (TE) = 2,300/2.98 ms, flip angle = 9°, field of view (FOV) = 256 × 256 mm2, matrix size = 256 × 256, slice thickness = 1 mm, no interslice gap, and 176 slices).

Area of acquisition: Whole brain

Diffusion MRI: ☒ Used ☐ Not used

Parameters: Diffusion weighted images (DWI) were obtained using spin an echo-based echo planar imaging sequence, including 30 volumes with diffusion gradients applied along 30 non-collinear directions (b = 1,000 s/mm2) and one volume without diffusion weighting (b = 0 s/mm2). Each volume consisted of 45 contiguous axial slices (TR/TE = 6,100 ms/93 ms, flip angle = 90, FOV = 240 × 240 mm2, matrix size = 256 × 256).

Preprocessing

Preprocessing software: DPABI, MRtrix3, FSL

Normalization: Spatially normalized to Montreal Neurologic Institute (MNI) space, and re-sampled to 3 × 3 × 3 mm3 voxels

Normalization template: MNI305

Noise and artifact removal

The nuisance variables including the Friston 24-parameter model, ventricular signal, and white matter signal were regressed out.

Volume censoring

Frame-wise displacement (FD) was calculated for each time point. Participants were excluded if any of the following three criteria were satisfied, (i) mean FD exceeded 0.5 mm, (ii) head motion exceeded 3 mm or 3°, or (iii) more than 20% of all time points had FD values exceeding 0.5 mm.

Statistical modeling & inference

Model type and settings

The nonlinear stochastic differential equations of neural activity in each cortical region followed the same parameter settings as in the previous study (Kong et al., 2021, Nature Communication).

Effect(s) tested

We tested the spatial correspondence between the gradient of time-series features with microcircuit simulations by pMFM using Spearman's correlation coefficient.

Specify type of analysis: ☒ Whole brain ☐ ROI-based ☐ Both

Statistic type for inference

(See Eklund et al. 2016)

Parcel-wise

Correction

FDR, permutation, spatial autocorrelation.

Models & analysis

n/a	Involved in the study
☐	☒ Functional and/or effective connectivity
☒	☐ Graph analysis
☐	☒ Multivariate modeling or predictive analysis

Functional and/or effective connectivity

Pearson correlation

Multivariate modeling and predictive analysis

principal component analysis, diffusion map embedding to conduct the dimension reduction.
